# Erdheim-Chester Disease With Concurrent Lung Adenocarcinoma

**DOI:** 10.1177/2324709620918101

**Published:** 2020-05-02

**Authors:** Nadia Solomon, Arne Landwehr, Zerwa Farooq, Garfield Gutzmore, Horace Tang, Sandeep Singh Tuli, Vincent Rizzo

**Affiliations:** 1 St. George’s University, True Blue, Grenada; 2 Icahn School of Medicine at Mount Sinai, Queens Hospital Center, Jamaica, NY, USA

**Keywords:** cancer, challenges, communication, medical decision making

## Abstract

Erdheim-Chester disease (ECD) is a rare type of blood cancer characterized by infiltration of the body tissues by pathologic histiocytes, leading to widespread inflammation and damage. Clinical presentations range widely, from asymptomatic infiltration of bone to multiple organ system damage and resultant dysfunction. This report describes a case of a patient with several unusual imaging findings that led to a differential diagnosis of ECD; however, a biopsy of a mediastinal mass suspected to be due to histiocyte infiltration instead revealed primary lung cancer. Ultimately, ECD could not be ruled out, and the patient was referred to dermatology for a superficial facial xanthelasma biopsy, results of which were consistent with ECD. Concurrent ECD and adenocarcinoma is highly unusual; this case demonstrates the importance of a thorough investigation and the consideration that not all findings may be attributable to a single disease process, even when the alternative is very unlikely.

## Introduction

Erdheim-Chester disease (ECD) is a rare, non-Langerhans’ cell histiocytosis, with only several hundred cases appearing in the literature.^1,2^ Originally described as “lipoid granulomatosis,”^3^ ECD is characterized by tissue infiltration by pathologic, foamy histiocytes, leading to inflammation and damage.^1,4^ More than 50% of patients demonstrate mutations in the proto-oncogene BRAF—a serine/threonine protein kinase responsible for transducing mitotic signals from the cell membrane to the nucleus—leading to uncontrolled clonal expansion.^3,4^ Clinical manifestations range from asymptomatic infiltration of bone to multiple organ system damage and dysfunction.^1-3^

The following report describes a case of this uncommon disease, presenting in a patient with an additional cancer process that initially distracted from the diagnosis. This case also highlights the diversity of ECD in terms of its clinical presentation and affected organ systems, and discusses the utility of multiple imaging modalities in contributing to making the diagnosis, emphasizing the importance of a thorough radiological workup and high level of clinical suspicion.

## Case Report

**Figure 1.**
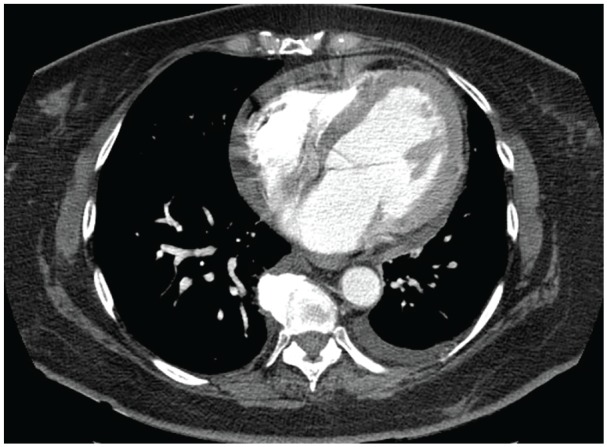
Axial contrast-enhanced computed tomography scan demonstrates small pericardial and left pleural effusions.

**Figure 2.**
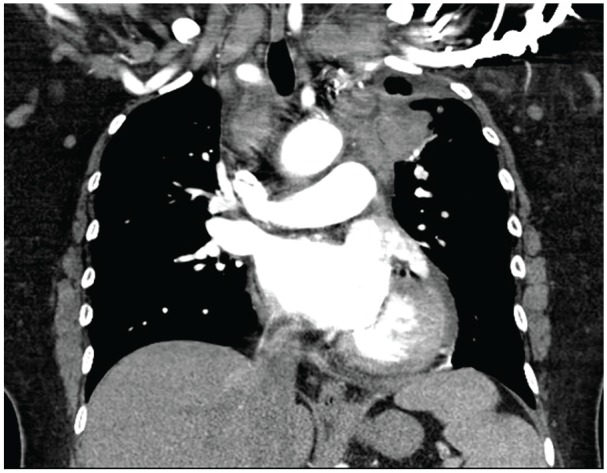
Contrast-enhanced coronal reformation of chest demonstrates a mass in left upper lobe, accompanied by mediastinal and bilateral supraclavicular lymphadenopathy.

**Figure 3.**
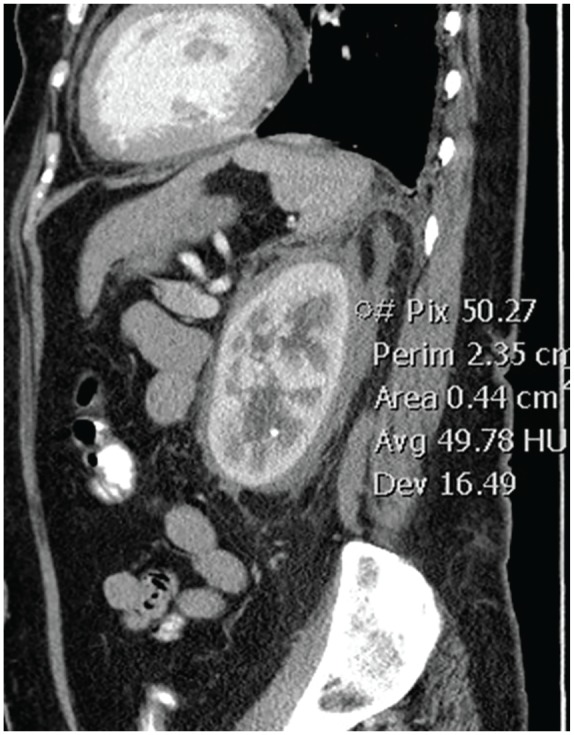
Sagittal computed tomography image through the left kidney with the circled region of interest demonstrating a density measurement of 49.78 Hounsfield units, confirming soft tissue infiltration of the perinephric space.

**Figure 4.**
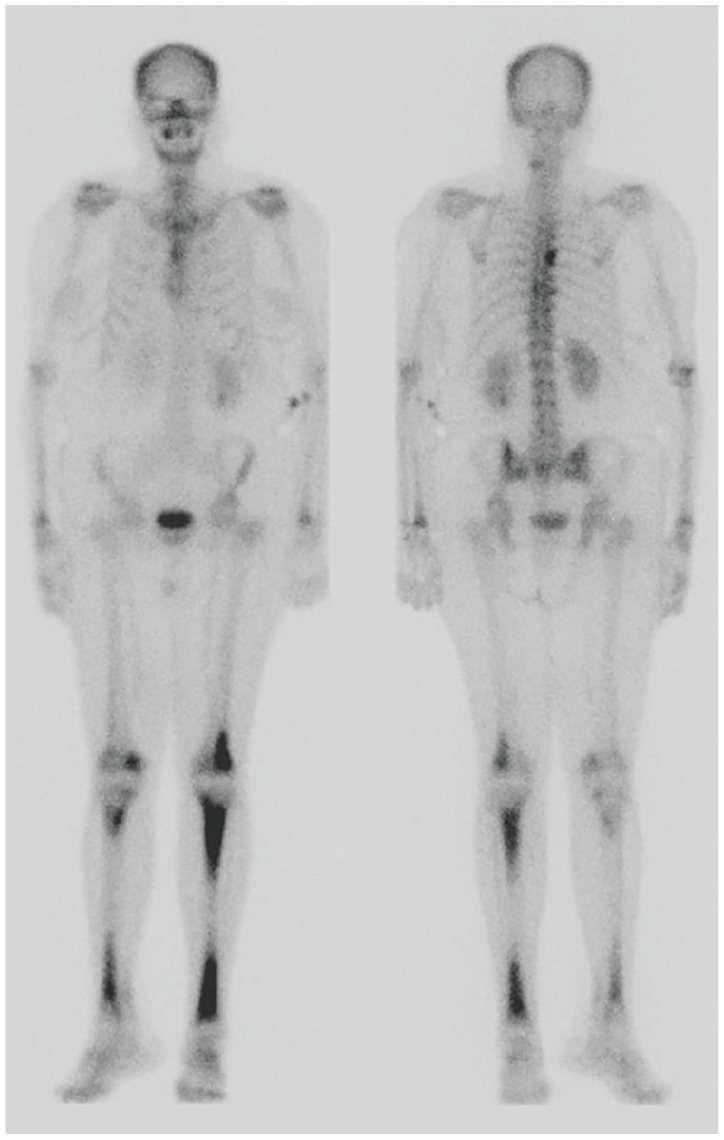
Anterior and posterior whole-body bone scan images demonstrate intense uptake in distal femurs and tibias, and to a lesser extent the radius distally in both arms. The predominant sites of involvement in the bone are the diametaphyseal regions. Uptake in the mid-thoracic spine, right of midline, was due to proliferative and abundant osteophytes, and uptake in the cervical spine was degenerative in nature.

A 56-year-old Trinidadian female presented to the emergency department with intermittent left-sided chest pain for 3 days, which was exacerbated by exertion and associated with shortness of breath. Additional symptoms included headache, fatigue, and 15- to 20-pound weight loss with bilateral lower extremity pain over the preceding year. Physical examination revealed numerous facial xanthelasmas. Chest X-ray revealed an enlarged cardiac silhouette, suspected pulmonary vascular congestion, and subcentimeter calcifications due to old granulomatous disease. Computed tomography (CT) of the chest revealed small pericardial and left pleural effusions (Figure 1), and a large left upper-lobe paramediastinal mass with extensive adenopathy (Figure 2), warranting metastatic workup. Subsequent CT of the abdomen revealed perinephric soft tissue surrounding both kidneys (Figure 3). CT head without and with intravenous contrast revealed metastases to the right frontal cortex and right posterior cerebellum with mild mass effect of the right lateral ventricle. Magnetic resonance imaging of the brain confirmed these findings, demonstrating enhancing lesions in the right cerebellum and right frontal lobe adjacent to the frontal operculum. A total-body bone scan revealed abnormal uptake in the seventh vertebral body and increased uptake in both lower extremities (Figure 4).

CT-guided biopsy of the superior mediastinal lymph nodes revealed poorly differentiated adenocarcinoma with necrosis positive for cytokeratins AE1/AE3 and TTF1, compatible with lung primary. Findings and suspicions were discussed with the patient, and she was referred to dermatology for biopsy of right cheek xanthelasma. This biopsy revealed a dermal nodular infiltrate of histiocytes with foamy cytoplasm mixed with lymphocytes and rare Touton-type giant cells, consistent with ECD.

## Discussion

Although definitive diagnosis of ECD relies on identification of CD68(+), CD1a(−) histiocytes on biopsy, radiological findings can suggest the diagnosis.^2^ Because ECD affects multiple organs and organ systems, however, presentation at diagnosis and associated imaging findings can be highly variable. In this case, demonstration of perinephric soft tissue encapsulating the kidneys on CT and giving them a “hairy” appearance was the first sign alerting clinicians to the potential presence of ECD. CT findings of pericardial and pleural effusions,^2^ combined with findings of increased uptake in the lower extremities on bone scan,^1^ further supported this diagnosis. As central nervous system involvement, often characterized by multiple lesions, has been reported in >50% of patients.^1,2,5^ The 2 brain lesions were also considered consistent with the working diagnosis. It was therefore somewhat surprising when the mediastinal lesion was biopsy proven as adenocarcinoma, with ECD proven later on via xanthelasma biopsy.

Concurrent diagnosis of ECD with adenocarcinoma is rarely seen: a PubMed search using the terms “Erdheim Chester cancer,” “Erdheim Chester carcinoma,” and “Erdheim Chester adenocarcinoma” revealed no other reported cases of ECD co-occurring with adenocarcinoma, and only one case of Erdheim-Chester co-occurring with papillary thyroid carcinoma.^6^ Given confirmation of the presence of both types of cancer, it is unclear if the central nervous system lesions are due to ECD or to metastatic spread of adenocarcinoma.

It is estimated that only a quarter to half of patients with ECD present with cutaneous manifestations of the disease^1,7^; a recent study by Chasset et al identified xanthelasma-like lesions as the most common cutaneous manifestations (25% of cases).^8^ As such, it was unclear if the biopsy would indeed yield positive results or if the patient’s facial xanthelasma would be due to other causes. Given the multitude and diversity of what were highly suspected to be ECD manifestations in this patient, suspicions that the facial lesions would demonstrate the same etiology were also high; however, with limited information available on how frequently xanthelasmas in ECD patients are in fact due to the disease, this potential downfall of the biopsy was emphasized when the option was presented to the patient. Finding a positive result was therefore encouraging; it raises the question of how specific this sign may be for the disease, since a facial xanthelasma biopsy, if possible, would be a less invasive and low-risk diagnostic option for patients with xanthelasma suspected to have ECD.

Erdheim-Chester disease should be considered as a differential diagnosis in patients with signs of malignancy accompanied by diffuse tissue thickening and fibrosis. Simultaneous occurrence of ECD and adenocarcinoma is highly unusual; in this case, the presence of adenocarcinoma was biopsy confirmed, but could not explain the majority of imaging findings. Xanthelasma biopsy may be a viable option to diagnose ECD, but it may be beneficial to further investigate the specificity of this sign in this patient population. Ultimately, this case demonstrates the importance of a thorough workup: in a disease like ECD with such diverse features, it is important to consider that not all findings may be attributable to a single disease process.
